# Obstetrics

**Published:** 1892-12

**Authors:** L. M. Griffiths


### OBSTETRICS.

The termination of a tedious labour by forceps is usually such a relief both to patient and attendant that it is not a matter of wonder if sometimes sufficient attention is not given to possible injury which may be inflicted on mother and child. Much has been, and is still being, written on this subject. No more excellent summary of the whole question is to be found than that in the *American System of Obstetrics.*[1] Dr. J. G. Swayne, recently calling attention[2] to some of these dangers, refers to the often overlooked risk of compressing the cord in cases where it is coiled round the neck, and advises the operator to be careful not to pass either blade of the forceps unnecessarily far.

A new interest for English readers[3] is given to obstetric traumatisms by Dr. George H. Rohé, of Baltimore, who, in a paper at the June meeting of the American Medical Association,[4] stated his belief that puerperal mania often takes its origin from some lesion of the genital tract. He gave the details of some cases, from a consideration of which he felt justified in believing (1) that, in at least the majority of cases, puerperal insanity is an infective psychosis, (2) that careful observation

[2] *Bristol Med.-Chir. Journ.*, Sept., 1892, p. 158.

[3] Dr. Th. B. Hansen published in Denmark in 1888 a work on the relation between puerperal psychoses and puerperal infection.

[4] *Journ. Amer. Med. Assoc.*, July 16, 1892.

will show that few cases of puerperal insanity occur without a preceding or coincident puerperal infection. For this belief he gave the following reasons:

(1) Puerperal insanity occurs, in the great majority of cases, within the first ten days after delivery—about one-half in the first five days—the same period during which puerperal infection usually occurs.

(2) It is usually accompanied by elevation of temperature, and other evidences of febrile disturbance.

(3) The clinical form in which puerperal insanity manifests itself is, in the majority of cases, that of acute delusions or confusional mania. Depressive states are rare, except as secondary lesions. In other words, the most frequent condition is one most closely resembling febrile delirium.

(4) The death-rate is much higher than in simple mania. Death occurs from exhaustion, usually with high temperature and rapid pulse.

(5) *Post-mortem* examinations, though apparently infrequent in these cases, have shown grave involvement of the pelvic viscera.

(6) Examinations of the pelvic organs during life show lacerations of the perineum and cervix uteri (facile channels of infection in the puerperal woman). As secondary conditions are found, intra-pelvic (peritoneal) inflammation, and consequent abnormal locations, fixations, and congestions of the uterus, tubes, and ovaries.

(7) The results of operations seem to show that removal of local sources of irritation increases the chances of recovery from the mental disease.

A sufficient number of cases, showing cause and effect, has not yet been recorded; but Dr. Rohé's suggestions are valuable in directing attention to any possible factor which may give rise to this distressing affection, and they afford a reason for additional care in endeavouring to avoid the least parturient lesion of the uterus or vagina.

Independently confirmatory of Dr. Rohé's theories come the observations of Prof. Olshausen, who has called attention to the relation of psychoses to grave infectious disease of the puerperium. He considers that cases of puerperal pyæmia, with ulcerative endocarditis, are those that are usually followed by mental disease.[1]

[1] Abstract from *Zeit. f. Geburt. und Gyn.* in *Jour. Nerv. and Ment. Dis.* Sept., 1892, p. 725.

Traumatisms of the cervix deserve more consideration than they often receive. Fraisse[1] holds that every delivery at term in a primipara is accompanied by a laceration of the cervix. He arranges such injuries into four classes. Those of the first degree undergo immediate repair. In the second class, the laceration extends beyond the margins of the os, but stops at the vaginal insertion. In the third degree, which is of a very grave character, the wound may extend as far as the peritoneum. The fourth form is in reality a rupture of the uterus. Fraisse dwells with some detail upon the injuries of the third degree, which present the same symptoms and require the same treatment, whether their origin be art or nature. The first symptom is hemorrhage, which is differentiated from that caused by uterine inertia, by noting that the contractions and retraction of the uterus occur normally. Fraisse says that if the uterus is firmly contracted, and if there are no external lesions, any abundant hemorrhage may be assumed to have its origin in a laceration of the cervix which extends beyond the insertion of the vagina. In such a wound there are obvious dangers of an immediate nature. The hemorrhage may usually be stopped by pressure. Infection through the wound must be prevented. The remote results are familiar to gynæcologists, and manifest themselves by pain, inflammatory troubles, sterility, and abortion.

Without doubt the child often shares with its mother the disadvantage of receiving injuries which are either not observed or not manifested at the time. Dr. L. L. Gray, in calling attention[2] to some types of paralysis in young children, adds to the testimony already existing concerning the production of these by injuries due to difficult delivery or the use of instruments.

Quite a formidable list of ocular injuries received in forceps-delivery is given by a writer in the *Medical News* of April 16th, 1892. All the evidence, excessive but much disregarded, should make the obstetrician ever on his most acute guard to minimise the dangers which may arise to mother or child by the use of the forceps.

A curious note on unavoidable brain injuries is supplied by Dr. F. W. Goodall,[3] who attributes our right-handedness to a condition of left-handed weakness produced, through the frequency of the child presenting in the first or fourth position, by the right side of the head being subjected to

[1] *Nouv. Arch. d'Obst. et de Gyn.*, Feb., Mar., 1892, quoted in *Amer. Journ. Obst.*, Sept., 1892, p. 424.
[2] *Internat. Clin.*, vol. i., p. 204. [3] *Journ. Amer. Med. Assoc.*, July 4, 1891.

a greater degree of mechanical pressure and violence than is the left side.

Writing on "The Pelvic Symphyses in Pregnancy and Parturition," Dr. W. J. Conklin [1] supports the conclusions of Luschka, that these symphyses are true joints provided with synovial membranes, and capable of limited motion. Under the stimulus of pregnancy the softer structures of the symphyses undergo a general relaxation, which necessarily leads to a slight separation of the articulating bones. Dr. Conklin does not discuss the question of the mobility of the sacro-vertebral and sacro-coccygeal joints, as he believes it is admitted by all obstetricians. He adduces a goodly array of evidence in support of the view that there is during labour a considerable separation of the sacro-iliac symphysis and of the pelvic joints. Admitting that separation of the pelvic joints does take place to a greater or less extent in many, if not all, women at times, its value as an obstetrical factor depends wholly upon the extent of the separation. It becomes difficult to decide whether in some of the recorded cases the relaxation was normal or pathological. Probably the cases in which the separation was considerably over an inch were of the latter class. Dr. Conklin gives details of some of his own cases, in which the relaxation exceeded physiological bounds, and caused pain and disability, partial or total, of the lower extremities. He infers that many labours which, contrary to expectation based upon former experience, have proved to be short and easy, have been so on account of considerable relaxation of the symphyses during pregnancy, by which the pelvic circumference has been notably increased.

In the English 1890 edition of Winckel's *Midwifery*, a fervent wish is expressed that the operation of symphysiotomy should be for ever entombed. Recent experience shows that this wish is now farther than ever from being attained. The operation of separating the pubic bones at their junction with one another, usually called symphysiotomy,[2] was introduced before the Royal Academy of Surgery of Paris in 1768, by Sigault, who hoped that it would take the place of Cæsarian section. After some adverse criticism, the operation became for a time a popular one. It then became dis-

[1] *Amer. Journ. Obst.*, Nov., 1892.

[2] Dr. W. S. Forbes (*Med. News*, Oct. 29, 1892) rightly points out that in the sense used the word has no etymological justification, as it does not indicate the particular symphysis which is to be divided. He suggests "pubeotomy."

countenanced, and till its partial revival in Italy, about twenty-five years ago, had passed out of obstetric practice. It seems likely, however, that the operation will become a recognised one, and it promises under antiseptic precautions to be a remarkable success. The credit of this is mainly due to Prof. Morisani, of Naples. Dr. Robert P. Harris gives [1] a full review of the history of the operation, and its mode of performance. From Jan. 1st to Oct. 8th, 1892, there were 52 operations, with one death.[2] In the cases of recovery the unions were good, and there was no resulting lameness. Seven children were lost. Only one was born dead. Three were born in an asphyxiated condition, and the rest died during the first three days. If the child did not survive beyond the first three days it was counted as lost. Out of 52 children, 45 lived; 51 were born alive.

A. Pinard[3] considers that with antisepsis the operation is not only devoid of danger, but is beneficial. He says that the pubic bones can be separated to the extent of six centimetres, and that the operation itself is not difficult. Pinard is of opinion that in many cases this operation should take the place of embryotomy and of Cæsarian section, that the lives of many women and children may be preserved by it, and the practitioner be saved the cruel necessity of crushing the skulls of living infants.

Dr. W. T. Lusk, commenting on Dr. Harris's paper, said that it gave him great pleasure to find that symphysiotomy had been of recent years so far perfected that it gave a lower maternal death-rate than craniotomy.

Dr. B. C. Hirst[4] gives a summary of the statistics of the operation, which down to 1858 had a maternal mortality of 33 per cent.; from 1866, when it was again practised, till 1886 the mortality was 24 per cent. Dr. Hirst says the first operation in America was performed by Dr. Charles Jewett, of Brooklyn, on September 30th of this year. He considers the modern revival of the operation to be the most important advance in obstetric surgery since the general adoption of abdominal section in early extra-uterine pregnancy. Dr. Hirst gives the clinical records of some typical cases, including one in which he operated at the University Maternity of Philadelphia. The choice was between Cæsarian section, craniotomy, or symphysiotomy. Mother and child did well. Dr. Hirst gives details of the operation, which he considered

[1] *Amer. Journ. Obst.*, Oct., 1892.
[2] *Med. News*, Nov. 5, 1892, p. 528.
[3] Abstract in *Amer. Journ. Obst.*, July, 1892, p. 134, from *Annales de Gyn.*, Feb., 1892.
[4] *Med. News*, Oct. 15, 1892.

should be performed with Galbiati's knife. A fuller account of the mode of operation is given by Dr. Harris. Dr. Anna E. Broomall performed a similar operation at the Women's Hospital, Philadelphia, on October 7th. On October 14th mother and child were both doing well. In the discussion which followed the reports of the cases of Dr. Hirst and Dr. Anna Broomall it was pointed out that the introduction of antiseptics had revolutionised the operation. Its performance has now received the additional sanction of Charpentier and Porak of Paris, Leopold of Dresden, and Freund of Strassburg, all of whom have had successful cases.

Porak's first case is specially instructive. After making all justifiable attempts with the forceps, he failed to deliver the disproportionately large child. He did not think that the case was one for Cæsarian section. He opened the symphysis, put on the forceps a second time, and accomplished the delivery without trouble. The woman recovered, and the child lived.

On November 22nd, Dr. W. J. Smyly performed the operation at the Rotunda Hospital, Dublin. This is said to be the first symphysiotomy in the United Kingdom since 1782. Nine days after the operation mother and child were doing well.[1]

In the light of these recent successes, and of the fact that symphysiotomy has been chosen as a subject for discussion at the International Congress, it is of interest to note the views of the older obstetricians on the question. William Hunter, arguing from experiments on the cadaver not realising that after death the elasticity of the ligaments soon becomes lost, stated that the symphysis pubis could not be separated to the extent of an inch and a half without lacerating the sacro-iliac ligaments. Ryan cited "unanswerable objections to this highly dangerous and unwarrantable operation."[2] Ramsbotham says: "The operation is not justifiable in cases of the more deplorable distortions of the pelvis, nor in the smaller degrees of diminution, because in them craniotomy can be performed."[3] Matthews Duncan believed that there was a future for the operation, and entered a protest against the unfounded calumnies which had been directed against it by British authors, who "have raised difficulties about it which are sufficient to deter a superficial inquirer from its consideration."[4] He quotes the words of Denman, who,

[1] *Brit. Med. Journ.*, Dec. 3, 1892.
[2] *Manual of Midwifery*, 3rd ed., 1831, p. 598.
[3] *Obstetric Medicine and Surgery*, 2nd ed., 1844, p. 294.
[4] *Mechanism of Natural and Morbid Parturition*, 1875, p. 158.

although he spoke cautiously about the operation, did not "mean to insinuate a wish, or advance an argument in favour of this operation in the cases for which it was originally proposed." So quickly has the change of opinion come about in reference to this operation, that no longer ago than 1889 Dr. Robert P. Harris, now one of its most enthusiastic advocates, said that "symphysiotomy cannot take the place of the Cæsarian section and its modifications except to a very limited extent. It has a range of only a fraction over half an inch."[1]

L. M. GRIFFITHS.

### OTOLOGY.

[1] *System of Gynecology and Obstetrics.* Obstetrics, vol. ii., p. 279.

